# A mathematical model and experimental procedure to analyze the cognitive effects of audio frequency magnetic fields

**DOI:** 10.3389/fnhum.2023.1135511

**Published:** 2023-05-12

**Authors:** Enrique A. Navarro, Enrique Navarro-Modesto

**Affiliations:** ^1^Departament de Informàtica, ETSE, Universitat de València, València, Spain; ^2^Escola Politècnica Superior de Gandia, Universitat Politècnica de València, València, Spain

**Keywords:** environmental magnetic fields, audio frequency magnetic fields, transcranial magnetic stimulation, cognitive disorders, working memory, magnetic fields

## Abstract

Audio frequency magnetic fields (20 Hz−20 kHz) are magnetic fields in extremely low frequency-very low frequency (ELF-VLF) bands that are present near audio equipment and acoustic transducers. These devices transform and operate the electrical signal from the recordings or other devices into acoustic and audio signals. The cognitive influence of sound and noise has been widely studied and recognized since the times of ancient Rome; however, the cognitive effects of the magnetic fields of these frequencies have not been studied. Due to the extensive use of audio devices that use this type of transducer near the temporal–parietal area, we believe that it is of interest to study their impact on short-term memory or working memory (WM) and to analyze their potential as they operate as a transcranial magnetic stimulation. In this study, a mathematical model and an experimental tool are introduced to analyze memory performance. The model dissociates the reaction time of a cognitive task. We analyze the model in data from a group of 65 young, healthy subjects. WM is assessed in our experimental setup by means of the Sternberg test (ST), whereby during the ST, one subgroup was exposed to an audio frequency magnetic stimulus, and the other subgroup received a sham stimulus. The magnetic stimulus was ~0.1 μT and was applied to both sides of the head at the frontal cortex near the temporal–parietal area, which is where WM is expected to be located. The ST records reaction times when determining whether an object displayed on the computer screen is one of the objects to be remembered. The results are analyzed within the mathematical model and changes are observed, including the deterioration of WM, which could affect 32% of its operability.

## Strength

A simple framework to assess cognitive performance is presented. An audio-frequency magnetic field of very low intensity was used to stimulate the prefrontal cortex. The effects on the WM were dissociated. Results are statistically significant.

## Weakness

We do not address the specific underlying biophysical mechanism. The obtained results are for a very specific group of healthy young subjects; results may vary in a different group.

## 1. Introduction

Magnetic fields (MFs) in the audio frequency range (MF-Audio) are MFs with frequencies between 20 Hz and 20 kHz that are located near any electrical and electronic equipment that operates at these frequencies. MF-Audio are fields in the extremely low frequency-very low frequency (ELF-VLF) bands that are present near audio equipment and acoustic transducers. Audio equipment transforms the electrical signal from the recordings into an acoustic signal; their coverage is reduced because they are quasi-static fields that decay very quickly with distance, but in certain cases, the proximity of the user means this exposure should be taken into account. They are omnipresent due to the widespread use of audio systems and other electromechanical devices operating at these frequencies. In the home, these fields are generated by household appliances, such as washing machines, refrigerators, televisions, cell phones, monitors, computers, and energy-saving fluorescent lighting devices. Some of these systems have high spectral content emissions, in many cases, up to megahertz, and are aptly named “dirty electricity” (Havas, 2008). They are mainly generated by audio devices, and the extent of their exposure is determined by the proximity to the body of such devices, as in the case of loudspeakers and headphones. Portable digital music devices (MP3 players or equivalent) are widely used, with over 100 million units sold in 2007, and ~350 million as of September 2012 (Costello, 2014a,b). Depending on the model, these audio devices produce a magnetic induction near the temporal lobe of the brain of ~0.1 μT, 20 Hz−20 kHz. These devices can produce interference when placed near a pacemaker (Lee et al., 2009). Kilohertz signals are also used in PLC co-communications to control electricity and gas consumption in-smart meters (Galli et al., 2008) through electricity supply wires.

We believe that this exposure justifies research into low-intensity MF-Audio, ~0.1 μT, 20 Hz−20 kHz, to study whether there are any effects on the nervous system or whether there is any influence on behavior. Electromagnetic exposure protection standards were much more restrictive in Eastern countries than in Western countries before the 1990's, largely because of the effects detected on the nervous system by these exposures (Presman, 1970; Szmigielski, 1989). There is significant evidence in the scientific literature demonstrating the influence of MFs on behavior, motor activity, and neurotransmitters in the human brain (Trzeciak et al., 1993; Chance et al., 1995; Pesic et al., 2004). There are results from both human studies (Trimmel and Schweiger, 1998) and animal studies (Kavaliers et al., 1993, 1996); however, most existing research focuses on animal studies, analyzing memory and learning under the influence of MF exposure. It was shown that brief exposure to relatively weak MF could affect spatial learning and memory in rodents (Kavaliers et al., 1993, 1996). It was shown how an MF exposure of 60 Hz−0.75 mT prior to routine learning activities impaired spatial memory in laboratory rats, (Lai, 1996; Lai et al., 1998; Sienkiewicz et al., 1998; Lai and Carino, 1999). Further studies in rodents (McKay and Persinger, 2000) analyzed the effects of acute exposure to 2 mT−8 mT, showing effects on spatial memory consolidation and retrieval. MF stimulus of 3 μT and 60 Hz worsened a practice-associated improvement (Corbacio et al., 2011). Trimmel and Schweiger (1998) found an immediate reduction in cognitive performance under the influence of a 1 μT−50 Hz field in the head area. The effects of MF on cognitive performance were related to brain electrical activity (Bell et al., 1991; Marino et al., 1996, 2004; Dobson et al., 2000; Lyskov et al., 2001). The effects of MF proposed a possible mode of action through alterations in opioid activity (Schwartz et al., 1989; Kavaliers et al., 1993, 1996; Tolomeo et al., 2019).

Although a clear relationship between electromagnetic brain exposure and cognitive functions has not yet been established (Ikeda et al., 2019; Hu et al., 2021), it has been speculated by the Leone group that MFs interfere with neuropsychological processes (Ferrucci et al., 2008) responsible for short-term learning, supported by synaptic plasticity in the brain (Fregni et al., 2005). Maxwell's equations relate electric fields, magnetic fields, charges, and currents to inside the brain's biological material (Stratton, 1941; Kraus, 1999); a MF generates eddy currents, and electrical electrodes produce conduction or displacement currents, i.e., intracranial current stimulus, electric stimulus, or magnetic stimulus, which produce the movement of charge carriers with similar consequences depending on the intensity and frequency (Kraus, 1999).

There is a considerable lack of research on the effect of MFs of certain frequencies on the human brain and cognition (Lee et al., 2023), specifically on the effect of MFs on memory and behavior. This is due to the difficulties in covering the entire electromagnetic spectrum and all related technologies. Previous studies have mainly analyzed exposures to 50 Hz and 60 Hz, (Karimi et al., 2020) and radiofrequency communication bands (Freude et al., 1998; Hirata et al., 2021), but audio frequencies have never been studied. Headphones are audio transducers and generate an acoustic signal using an electromagnetic signal of the same frequency. The current carrying this signal is within the kHz band and is the source of the associated MF. These devices produce a low-intensity magnetic field near the areas of the brain involved in the processes associated with memory and especially short-term memory, often named working memory (WM), which is related to the parietal cortex of the brain, (Babiloni et al., 2004; Passingham and Sakai, 2004).

Short-term memory is a cognitive system with a limited capacity that can temporarily retain information for seconds (Miyake et al., 1999). WM is a broader and more important concept for reasoning and guiding decision-making and behavior (Cowan, 2008; Malenka et al., 2009; Diamond, 2013). WM is a central theoretical concept in cognitive psychology, neuropsychology, and neuroscience which was originally coined by Miller, Galanter, and Pribram (Pribram et al., 1960) in the 1960's in the context of theories comparing the mind to a computer. In Atkinson and Shiffrin (1968) used the term “short-term memory” to describe short-term storage. WM was previously named “short-term store” or “short-term memory,” “primary memory,” “immediate memory,” and “temporary memory” (Fuster, 1997); however, the ability to recall information for a few seconds would be better understood as a part or fraction of the broader concept of WM that lasts longer. Baddeley and Hitch (1974) introduced a multicomponent model for the functioning of WM (Baddeley, 1992). In general, it is recognized that WM has a limited capacity. One of the first quantifications was the “magic number seven” suggested by Miller (1956), who claimed that the information processing capacity of young adults is ~seven items, which he called “chunks,” regardless of whether the items are digits, letters, or words. Later, Cowan proposed that WM has a capacity of ~four chunks in young adults and less in children and older adults, (Cowan, 2001). Good WM performance is strongly related to performance on other complex cognitive tasks, such as reading comprehension, problem-solving, and IQ measures (Engle et al., 1999). WM capacity gradually increases throughout childhood (Gathercole et al., 2004) and gradually decreases in old age (Salthouse, 1994). WM is one of the cognitive functions most sensitive to decline with age (Park et al., 2002; Hertzog et al., 2003). Early WM degradation has been linked to the early onset of dementia and Alzheimer's disease; therefore, the assessment of WM seems to be a promising element for early diagnosis of cognitive impairment, as it is the first element of the cognitive structure of the brain (West, 1996; Gold et al., 2019).

The Sternberg item recognition paradigm or Sternberg test (ST) provides a computerizable timing method for the quantification of WM operability and its three respective components: perception, binary decision, and motor execution. The ST is a reaction time test that primarily dissociates motor and cognitive components in response times. The subject's responses are based on a temporally stored representation of the “chunks” or items to be maintained in the WM for the duration of the test. Sternberg (1966) showed that there is a linear relationship between response time (RT) and the number of items the subject must keep “on-line” in the WM. The slope of the linear function provides a measure of the cognitive component of response time. Response time increases linearly with each increase in WM load, i.e., items to be kept in the WM (Sternberg, 1966, 1969, 1975; Jensen and Lisman, 1998). In that response time (RT), there is a constant delay [the ordinate at the origin of the linear model (Sternberg, 1966)] that provides a measure of the time spent in perception, binary decision, and motor execution. The main functional stages can be characterized as (1) stimulus encoding; (2) serial memory scanning; (3) binary decision on the nature of the response; and (4) response organization and execution. In clinical studies, the ST has been widely used to assess attentional vulnerability (Robertson et al., 1997; Manly et al., 1999). Indeed, a population of patients with attention maintenance difficulties has been shown to exhibit frequent errors compared to normal controls (Bellgrove et al., 2006; Johnson et al., 2007).

There is scientific consensus about the difficulties in describing brain function and behavior as variables that allow for the assessment of behavior but are not sensitive enough to measure changes in brain functions (Kurokawa et al., 2003; Cook et al., 2006). The “Sternberg” paradigm is particularly sensitive and suitable for detecting minimal cognitive changes (Sternberg, 1966, 1969, 1975). Cognitive paradigms are always a difficult choice due to the lack of standardized norms and the fact that variations can be caused by, among other things, the choice of stimuli, timing, instructions given to the subject, and expected responses (Baddeley, 1992).

This study examines the immediate measurable effects on WM of MF-Audio applied to the area of the brain that sustains WM activity. The MF-Audio stimulus is ~0.1 μT in the kHz band. The intensity, frequency, and area of exposure would reproduce the equivalent MF exposure associated with loudspeakers and headphones. The effects on WM have been analyzed using the ST, under the exposure effect. A mathematical model has been proposed to analyze the impact on the reaction time (RT) of WM, which has been processed by multiple regressions to dissociate its motor and cognitive components. Working with a homogeneous group of 65 young, healthy volunteers, statistically significant results were obtained (*p* < 0.01). It was observed that exposure to MF-Audio produces a global reduction in reaction time of ~15 ms, related to perception, binary decision, and motor execution. However, a delay of 32% is obtained in the serial memory scanning process.

This study is novel and important in several respects; first, because of the frequency band of the MF explored, the MF-Audio band, which is in the kHz range. To our knowledge, there is no study by other authors that analyzes the effects of a continuous stimulus (CW) in the kHz band (1–20 kHz). There is also no other study by other authors that deals with the stimulation of the brain with such a low magnetic induction (0.1 μT). Second, this study addresses low-intensity exposure in young adults. Third, this study presents a convenient mathematical model with a computerized use of the Sternberg paradigm, which facilitates accurate synchronization to quantify the effects of MF-Audio exposure on the brain area supporting WM. The brain is the most delicate organ of living beings, and a procedure has been presented that facilitates the analysis of its subtle functioning in the handling of short-term information. This study presents a non-invasive and simple tool, with a mathematical model that dissociates the different parts of the cognitive process of WM. The results from a large group of young, healthy individuals are analyzed, and immediate effects are elucidated. Previous work has been done to study the cognitive effects of electromagnetic fields of other frequencies in humans and animals at 900-1800-2200-2400 MHz bands used in various communications (GSM, UMTS, 4G, WiFi); these studies have proliferated in the last 20 years (Hirata et al., 2021; Hu et al., 2021). Other studies have analyzed the effects of frequencies below 100 Hz (Blackman et al., 1987; Karimi et al., 2020; Lee et al., 2023). Studies on the cognitive effects of exposure to magnetic inductions at frequencies of 50Hz and 60Hz have proliferated (Liboff, 2019). The exposures to magnetic induction studied previously have been of the order of >1 μT, and most of the published studies focus on the mT range, which is between ten and ten thousand times greater than in the present study. All these studies have intended to question the ICNIRP protection guidelines for communications and power grid frequencies. There is also research on therapeutic applications for magnetic fields (Murphy et al., 2020), but other frequencies are mostly investigated therein (Liboff, 2019), using high magnetic inductions, between mT and Tesla, which is between 100 and 100,000 times the intensity we use. For instance, a magnetic induction of 20–150 μT-40 Hz was used in the study by Suszyński et al. (2014) for nerve regeneration, and 100 mT−20 Hz was used for neurophysiological treatments (Malavera et al., 2014; Gallasch et al., 2018) in transcranial magnetic stimulation (TMS) treatments in their different variants (Helekar et al., 2018; Kesikburun, 2022). Typical TMS therapies do apply magnetic induction pulses of several Tesla (Sparing and Mottaghy, 2008), which last <1 ms. The TMS pulse penetrates the skull and, in turn, induces short-term eddy currents in the brain tissue. TMS works with fields 100,000 times greater than the field that we use. Finally, our stimulus is a continuous wave (CW) field that lasts ~5–10 min. Therefore, our study also presents a new procedure for the magnetic stimulation of the brain, using a sinusoidal stimulus of kHz, with an extremely low but measurable magnetic induction.

## 2. Mathematical modeling

### 2.1. Statistical model

In our computerized model of the ST, the subject is asked to memorize a set of letters, after which the subject is shown letters, one at a time, and is then asked to decide whether these letters are the ones in the memorized set or not. It is reasonable to expect that the more items (letters) there are to recall, the longer it takes the brain to compare the displayed symbol with the memorized symbols. In other words, if the brain works sequentially in working memory, i.e., serially, not in parallel, it will spend more time retrieving symbols in WM. If the displayed symbol does not belong to the set to be remembered, the brain will serially scan the memory and reach the end of the set size (SZ), which is the number of letters to be remembered. However, if the displayed letter belongs to the memo-recalled set, we say that the letter is the target (TG case); the brain will stop searching when it finds it, and this will consume less time.

We do not know the exact position of each memorized symbol in the WM, we can only assume that the reaction time will be lower in the TG case. The proposed mathematical model of multiple regressions is, following Sternberg's work, (Sternberg, 1966, 1969) as follows:


(1) $$\begin{array}{l} \text{RT}=\alpha_{0}+\alpha_{1}\cdot \text{SZ}+\alpha_{2}\cdot \text{TG}, \end{array}$$

RT: Reaction time.

α_0_: Time spent in the preparation and execution of the response.

α_1_: Time used by the brain to search for a symbol. If the set size is one (SZ = 1), must remember one symbol and RT will increase α_1_, if SZ = 2 must remember two symbols, the RT will increase by 2α_1_, and so on. The reaction time RT depends on the number of symbols to remember, so we say that it increases as a function of SZ, and we add α_1_·SZ. The parameter α_1_ is the slope of the linear Sternberg model.

α_2_: Increase or decrease of the RT depending on whether the symbol is TG (TG = 1) or not TG, (TG = 0). It is an extra time that depends on whether it is a symbol to memorize or not. This extra time will be quantified as α_2_TG, being TG = 1/0.

If the stimulus affects WM, the reaction time RT of the model expressed in Equation (1) will change, and a new model is proposed. The new model follows Equation (2):


(2) $$\begin{array}{l} \text{RT}=\beta_{0}+\beta_{1}\cdot \text{SZ}+\left(\beta_{2}+\beta_{3}\text{SZ}\right)\cdot \text{EX}+\beta_{4}\cdot \text{TG} \end{array}$$

The difference in (2) with (1) is the introduction of the stimulus with the binary variable EX. If there is a stimulus, EX = 1, otherwise, EX = 0.

Equation (2) becomes Equation (1) for the case EX = 0:

α_0_ = β_0_

α_1_ = β_1_

α_2_ = β_4_

When there is a stimulus, EX = 1, there would be an increase in RT in the preparation and execution of the task due to the stimulus, which would be β_2_. Thus, we would have ΔRT = β_2_.

Moreover, in the case of stimulus (EX = 1), we hypothesize a delay in the memory search, which would depend on the number of symbols to memorize; this delay would be β_3_ for each symbol. The delay related to the number of items would be β_3_·SZ.

The mathematical model of equation (2) with stimulus, EX = 1, would result in a total RT delay = β_2_+β_3_·SZ.

### 2.2. Numerical model

A numeric-mathematical model using finite differences similar to previous studies (Soriano et al., 2004; Cepeda Rubio et al., 2011) was developed to calculate the MF levels produced by the two solenoids inside the head. The levels of MF in space were calculated by discretizing the Biot–Savart equation (Stratton, 1941; Kraus, 1999):


(3) $$\vec{B}(\vec{r})=\frac{\mu_{0}I}{4\pi }\int \frac{\vec{dl}\times (\vec{r}-{\vec{r}}^{\prime })}{|\vec{r}-{\vec{r}}^{\prime }{|}^{3}}$$

The above equation was discretized to numerically calculate ***B*** **=** (B_x_, B_y_, B_z_) in a three-dimensional space around the two solenoids. The vectors are now indicated in bold type:


(4) $$B(r)\;=\;\frac{\mu_{0}}{4\pi }\sum_{i1=1}^{N1}\frac{Il_{i1}\times \text{(}r-r_{i1}^{\prime })}{{\left|r-r_{i1}^{\prime }\right|}^{3}}\;+\frac{\mu_{0}I}{4\pi }\sum_{i2=1}^{N2}\frac{Il_{i2}\times \text{(}r-r_{i2}^{\prime })}{{\left|r-r_{i2}^{\prime }\right|}^{3}}\;\left(4\right)$$

The ***B*** field is calculated at each point of space ***r*** **=** (x_j_, y_j_, z_j_), by doing the numerical integration as a summation of the ***B*** field created by each small current I**Δ*l***_***i*1**_, I**Δ*l***_***i*2**_, located along the solenoid wires at ***r'***_***i*1**_ **=** **(**x'_i1_, y'_i1_, z' _i1_**)**, and ***r'***_***i*2**_ **=** ***(***x'_i2_, y'_i2_, z'_i2_).

In the numerical model:


(5) $$\begin{array}{l} I\;=\;\frac{V}{\sqrt{R^{2}+{\left(L\omega -1/C\omega \right)}^{2}}} \end{array}$$

μ__0__, Permeability of the free space (vacuum) 1.257 x 10^−6^ (Henry/m).

**Δ*l***_***i*1**_ **=** **Δ*l***_***i*2**_ **=** (Δl_x_, Δl_y_, Δl_z_). Path vector along the solenoid wires.

***r* =** (x_j_, y_j_, z_j_). Position of the point where ***B*** is calculated.

***r'***_***i*1**_ **=** **(**x'_i1_, y'_i1_, z' _i1_**)**. Position of ***dl***_***i*1**_.

***r'***_***i*2**_ **=** ***(***x'_i2_, y'_i2_, z'_i2_). Position of ***dl***_***i*2**_.

*R*. Resistance of solenoids (Ohm).

*V*. Output voltage of the generator (Volts).

ω = 2π*f* , (*rad/s*) being *f* the frequency (Hertz).

*L*. Inductance of the solenoid (Henry).

*C*. Parasitic capacitance of the solenoid (Farad).

**Figure 1:**
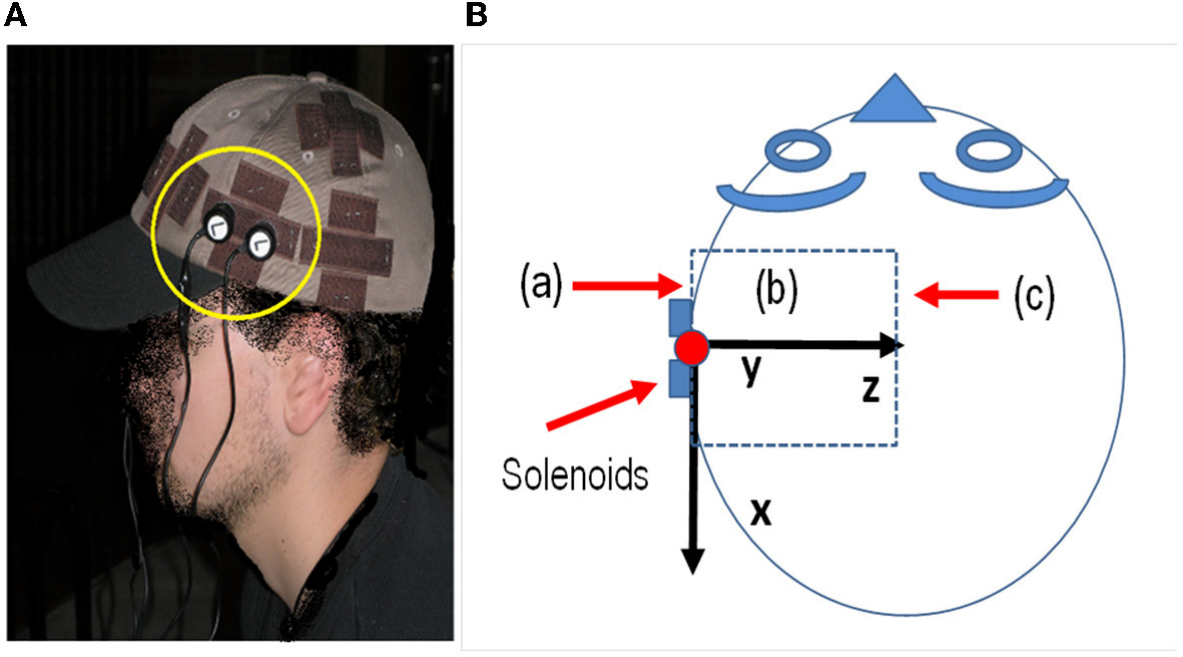
**(A)** Position of the electrodes in the experiment and area of the cortex with exposure. **(B)** Scheme of the head and x, y, and z axes relative to the subject's head, the position of the solenoids, and the volume where B is calculated, indicating planes (a), (b), and (c) where the magnitude of B is plotted in Figure 2.

**Figure 2:**
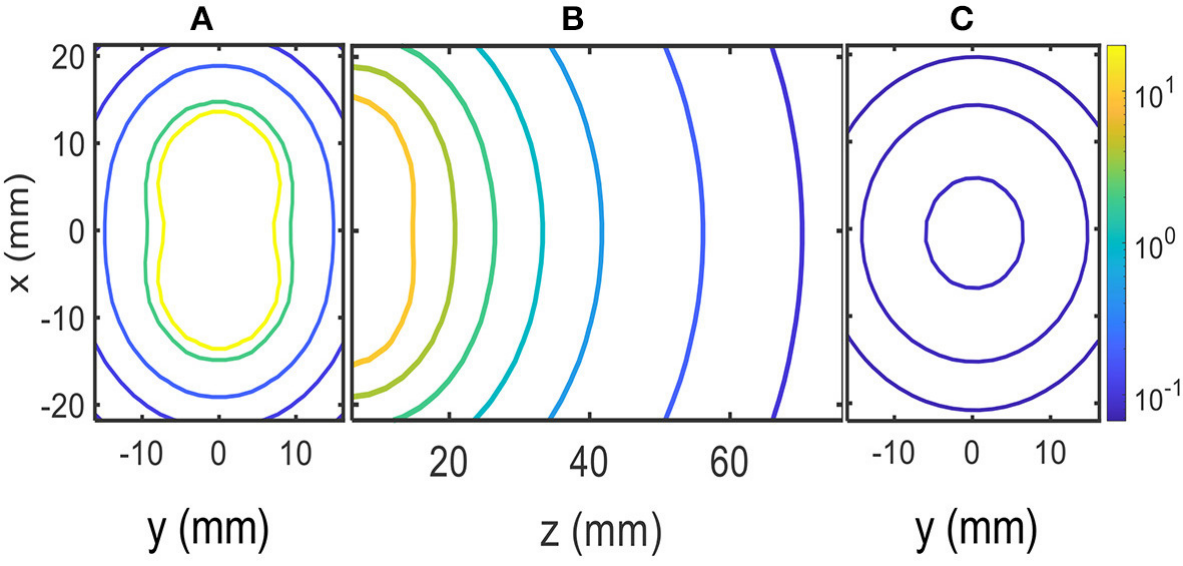
MF contour plots in μT units: **(A)** x–y plane at the edge of solenoids (z = 0). **(B)** z–x plane inside the head. **(C)** x–y plane z = 70 mm.

The above equations were used with the Matlab^TM^ software (MathWorks, 2023) to calculate the MF inside the head using the data of the solenoids and signal generator of the sound card. The solenoids (6.5 mm long and 7 mm average diameter) with 800 turns of AWG-40 copper wire provided a total resistance of 96 ohms and a total inductance of 4.7 mH. The magnetic field applied with the solenoids was deduced from the above equations using the voltage generated by the sound card and the impedance of the solenoids. Figure 1 shows the position of solenoids in the head of the subject and the three-dimensional volume where the ***B*** field is calculated. Figure 1A shows a picture of the actual setup of the experiment. Figure 1B shows a schematic of the head, a scheme of the x, y, and z axes relative to the subject's head, the position of the solenoids, and the volume where ***B*** is calculated, indicating planes (a), (b), and (c) where the magnitude of ***B*** is plotted in Figure 2.

Figure 2 shows the numerical results of the magnitude of ***B*** at several planes inside the head; these planes are shown in the scheme of Figure 1B. Contour levels in micro Tesla units (μT) are presented in Figure 2 with a color code in the logarithmic scale. The positions along the x–y–z axes are given in millimeters (mm). Figure 2A shows contour levels in the x–y plane at the edge of the solenoids close to the skull (z = 0). Figure 2C shows contour levels in the x–y plane at 70 mm from the edge of the solenoids inside the head (z = 70 mm). The calculated MF was ~0.10 μT at 70 mm from the edge of the solenoids, as shown in Figure 2C. Finally, Figure 2B shows contour levels in the x–z plane; it shows how the MF decays with distance to the solenoids along the z-axis inside the head, and contour levels approach 0.1μT near z = 70 mm.

## 3. Experiment

Sixty-five healthy adults were recruited for the study. Written informed consent was obtained from all participants prior to engaging in the study (Navarro et al., 2016). A signal in the 2–20 kHz band was generated using the sound card of a laptop computer. The sound card cable was routed in parallel through two pairs of copper wires to two pairs of solenoids. Each pair of solenoids was attached with Velcro fasteners to each side of a cloth cap worn by each test subject (see Figure 1). The four solenoids were symmetrically located on both temporal–parietal sides of the head, attached to the cap. The position of the laptop was away from the position of the volunteer, who was seated in a chair in a large classroom. The position was at least 10 meters away from the walls. The classroom was acoustically isolated with bricks and foam materials on the walls. Two solenoids were used to introduce the magnetic field.

The solenoids were attached to the cap, and after accounting for air and cap tissue, the edges of the solenoids were ~1 cm away from the skull. Our calculations and measurements with the EFA-300 probe B-100 cm^2^ meter (Narda Microwave, Hauppauge, NY, USA) show a magnetic inductance of ~0.10 μT within the cortex, at the position of the solenoids on the skull; however, other parts of the cortex were under similar or higher levels following the field lines of the solenoid on both sides of the head, as shown in Figure 2.

The background magnetic field in the environment, excluding our stimulus, was measured with the EFA-300 probe B-100 cm^2^ (original: ‘The background magnetic field, the one in the environment except for our stimulus, was measured with the EFA-300 probe B-100 cm2'). The measurement was less than 13 nT (nano Tesla) in the chair where the subject sat. The background level of the measured MF is the combination of the magnetic fields from natural sources and the EFA-300 meter's own internal noise, which has a detection limit of 10 nT. The magnetic field at the edge of the solenoids was also measured concordant with the calculations.

The stimulus was a continuous wave signal in the 2–20 kHz band. The signal was generated using the sound card of an HP Compaq nc6120 laptop computer managed with Cool Edit Pro software (Syntrillium Cool Edit Pro 2.0 Audio Editing Software, Adobe Systems Inc., San Jose, CA, USA). The signal was turned on at the beginning of the Sternberg test (ST) and turned off at the end of the ST. The duration coincided with the duration of ST, which was ~11 min.

**Figure 3:**
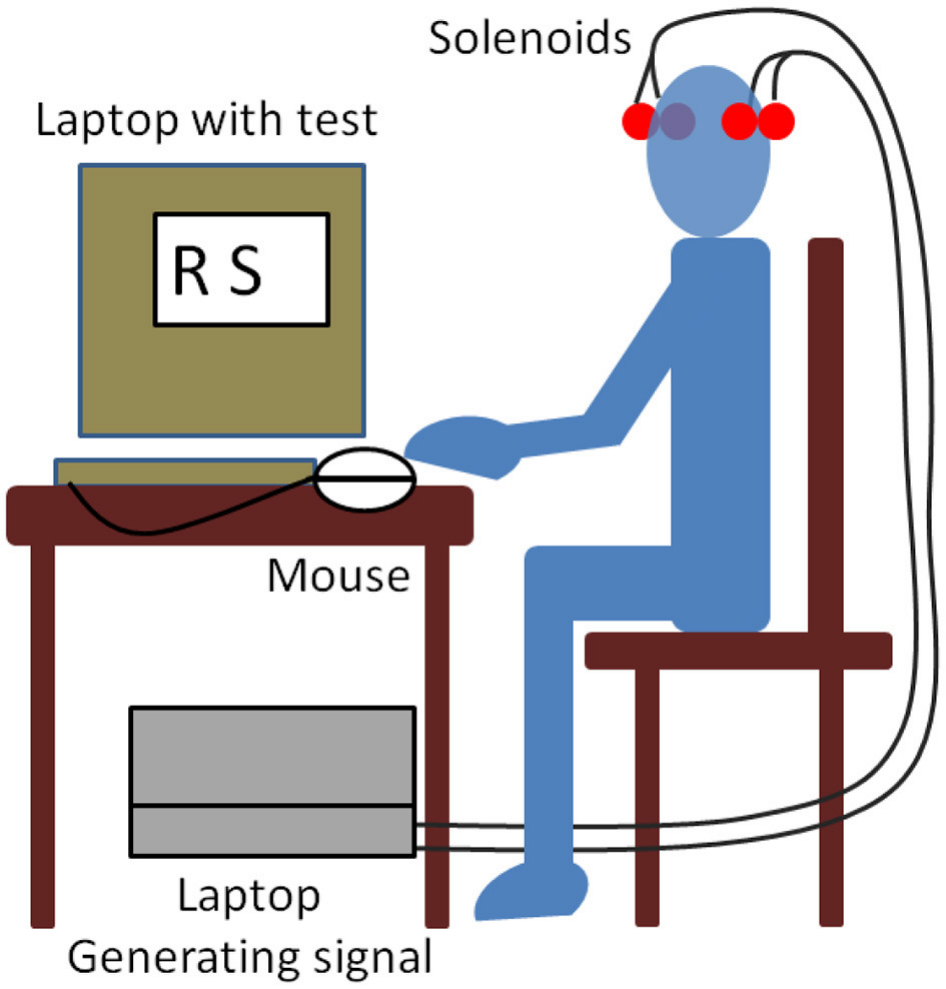
Experimental setup scheme.

The laptop used for the stimulus and the laptop used for the ST were battery-operated to minimize the presence of MFs. Ambient noise was measured with a sound level meter (CESVA™ SC-30) during each test. The average noise level during all tests was below 45 dB. The nearest noise source was a street with little traffic ~50 m away from the classroom. The test site was located at a distance of ~9 m from any electrically conducting wires. Illumination was from natural sunlight. All tests were conducted between 12:00 h and 13:00 h; a scheme is shown in Figure 3.

An Easynote laptop computer (Packard Bell BVEurope, Hertogenbosch, Netherlands) was used to present the information and record the subjects' responses. The items to be memorized were presented on the laptop computer screen (dimensions 28.5 cm × 21.5 cm) in the arial font (3 cm high) and viewed at a distance of ~60 cm. Responses were given using two hand keys on the mouse. The mouse was on the preferred side (right or left); the key for positive decisions corresponded to the right-hand side of the mouse, and the key for negative decisions corresponded to the left-hand side of the mouse. The symbols that the test subject had to memorize were displayed on the screen for 5 s. After displaying them, other symbols were shown on the screen for a maximum of 1 s, and the subject had to decide as quickly as possible whether those symbols were the ones they had to remember, in which case, they had to press the right side of the mouse, or not, in which case, they had to press the left side of the mouse. Each symbol appeared one after the other and remained on the screen for a maximum of 1 s. If the mouse button was pressed in less than 1 s, it disappeared and the next one appeared. If no button was pressed after 1 s, it disappeared and the next symbol appeared.

The RT was recorded in the computer as the time elapsed from the time the symbol was displayed on the screen to the time the mouse key was pressed. The total symbol set consisted of 21 consonants of the alphabet, shown in upper case. The set of symbols to be remembered (TG = 1) consisted of nine different letters, and the set of symbols that were not the ones to be remembered (TG = 0) contained 12 different letters. The ST was performed for each subject with 1-2-3-4 symbols (SZ = 1, 2, 3, 4), and the subjects were asked to respond as quickly and accurately as possible.

The exposure or sham-exposure was double-blind, neither the subjects nor the experimenters knew, and there were 34 subjects exposed to the MF stimulus (EX = 1) and 31 sham-exposed (EX = 0).

For each individual, within the ST, and for each SZ, we proceeded with a training phase, the subjects performed the ST in at least six practice attempts, so that the ST started to count when there were six error-free attempts. This was done so that the subjects could practice the ST, which they were performing for the first time, and at the same time, it prevented those with excellent WM from performing unnecessary training that could bias the study.

After the practice phase, we proceeded with the ST (SZ = 1, 2, 3, 4) with 12 tests for each SZ, i.e., a total of 48 tests. In total, each subject had 24 positive cases, TG = 1, and 24 negative cases TG = 0, which were randomly distributed during the 48 tests, and no more than three TG = 0 or TG = 1 came out consecutively.

## 4. Results

Demographic data and data regarding the participants' perceptions of their health were collected. We asked about common symptoms of poor health and discomfort: sleep quality; headaches; dizziness; vision and hearing disturbances; nervousness; sadness; and joint, skin, or digestive system problems. The ratings that were permitted to be marked were 0 deficient, 1 acceptable, 2 good, 3 very good, and 4 excellent. They were also asked if they received medication. Finally, they were also asked about their habits regarding daily presence in front of a computer screen, time spent daily using a personal computer, time spent daily playing video games, and minutes of daily use of a cell phone. They were also asked about tobacco consumption. Subjects who reported medication, tobacco consumption, or any illnesses were excluded from the experiment.

The subjects were randomly divided into two groups; the program decided who was exposed and who was not. This left one group with 34 subjects and the other with 31 subjects. Both the groups were analyzed *a posteriori* to analyze differences. The data were compared to determine whether the two groups were different. The health and demographic data are summarized in Table 1, giving average parameters and standard deviation for both groups. The parameters regarding their habits are compiled in Table 2, which also includes the average values and standard deviations in both groups. A two-sample *t*-test with unequal variance was used to test the null hypothesis of having a different mean. Differences were not found to be statistically significant. The means of the demographic, health, and habit data were analyzed by the *t*-test, giving non-significant results (*p* > 0.05). These results are shown in the last column of the tables, where it can be seen how the 95% CI for the mean contains zero in all cases. The same can be said for the habits reported in Table 2. It is not concluded that both groups have different means, as the CI includes both positive and negative values. Table 1 shows an acceptable health perception and homogeneity between both groups; and Table 2 shows homogeneity in habits between both groups.

**Table 1:** Demographical and health status for exposed and non-exposed subgroups. Mean with standard deviation. Differences between groups are not significant (*p* < 0.05).

**Subject**	**Sham-exposed (*n* = 31)**	**Exposed (*n* = 34)**	**Mean difference CI 95%**
Age	22.8 (2.5)	23.6 (2.3)	(−2.0,0.4), *p* = 0.17
Body mass index, kg/m^2^	25.1 (3.7)	25.8 (4.2)	(−2.7,1.2), *p* = 0.46
Headache	1.2 (0.7)	1.2 (0.8)	(−0.3, 0.4), *p* = 0.68
Sleep problems	1.2 (0.8)	0.9 (0.9)	(-0.1, 0.8), *p* = 0.12
Tiredness	1.4 (0.7)	1.4 (0.9)	(−0.5, 0.4), *p* = 0.86
Restlessness	1.0 (0.9)	0.8 (0.8)	(−0.2, 0.6), *p* = 0.37
Difficulty concentration	1.4 (0.9)	1.5 (0.9)	(−0.5, 0.4), *p* = 0.86
Joint pain	0.9 (0.8)	0.8 (0.9)	(−0.4, 0.5), *p* = 0.83
Nervousness	1.4 (1.2)	1.2 (1.0)	(−0.3, 0.8), *p* = 0.42
Nauseas	0.2 (0.5)	0.2 (0.4)	(−0.1, 0.3), *p* = 0.50
Lack Appetite	0.4 (0.5)	0.3 (0.5)	(−0.2, 0.4), *p* = 0.47
Feeling Sad	0.4 (0.8)	0.4 (0.6)	(−0.3, 0.4), *p* = 0.74
Loss Memory	0.6 (0.8)	0.5 (0.9)	(−0.3, 0.5), *p* = 0.63
Skin Problems	0.5 (0.8)	0.3 (0.5)	(−0.1, 0.6), *p* = 0.16
Visual Problems	0.4 (0.7)	0.2 (0.5)	(−0.1, 0.5), *p* = 0.15
Hearing Problems	0.3 (0.6)	0.1 (0.2)	(0.0, 0.4), *p* = 0.07
Dizziness	0.2 (0.5)	0.2 (0.5)	(−0.2, 0.3), *p* = 0.53
Cardiovascular problems	0.0 (0.2)	0.2 (0.5)	(−0.3, 0.0), *p* = 0.14
Health status	2.7 (0.7)	2.9 (0.76)	(−0.5, 0.1), *p* = 0.24

**Table 2:** Parameters regarding habits of subjects: Mean with standard deviation. Differences between subgroups are not significant (*p* < 0.05).

**Habits**	**Sham-exposed (*n* = 31 )**	**Exposed (*n* = 34)**	**Mean difference CI 95%**
Personal computer (hours/day)	4.8 (2.5)	4.0 (2.3)	(-0.4, 2.0), p = 0.18
Video games (1, 0)	0.7 (0.5)	0.6 (0.5)	(−0.2, 0.3), *p* = 0.61
Use of cellular phone (minutes/day)	7.1 (0.6)	7.1 (0.8)	(−0.3, 0.4), *p* = 0.75

The subjects performed the experiment following a particular order, in which exposed and unexposed subjects were randomly interleaved. The results for each subject were stored in a file with the initials of the name of the subject and whether the subject was exposed or not. The RTs in milliseconds for each case TG = 0, 1 and SZ = 1, 2, 3, and 4 were stored in this file. The number of mouse typing errors, or omissions if nothing was clicked, was also saved. The results are described in the following paragraphs. The data files were processed with Matlab^TM^ (MathWorks, 2023), and the statistical tests and the mathematical model used in the present study were performed using Matlab^TM^.

Errors and omissions in clicking the mouse of exposed and sham-exposed are shown in Table 3. The number of errors is low, with a mean of 7 for exposed and 8 for unexposed, a small difference, and we applied a *t*-test to check the differences. The results are not significant for the null hypothesis of having the same mean (*p* > 0.05). If we analyze the 95% CI of the difference between means, its value is in the interval (−1.8, 0.8), which contains zero, indicating that there are no significant differences in terms of errors. The same happens with the omissions in which the difference between means is in the interval (−0.4, 0.1) 95% CI, *p* > 0.05. It follows from these results, although not statistically significant, that exposure does not influence the number of errors and omissions.

**Table 3:** Average number of omissions (subjects do not press the mouse) and errors (subject press incorrectly the mouse) for the exposed and control subgroups: average and standard deviation.

**Type of error**	**Sham-exposed (*n* = 31 )**	**Exposed (*n* = 34)**	**Mean difference CI 95%**
Errors	7 (6)	8 (6)	(−1.8, 0.8), *p* = 0.45
Omissions	1 (1)	1 (1)	(−0.4, 0.1), *p* = 0.41

**Figure 4:**
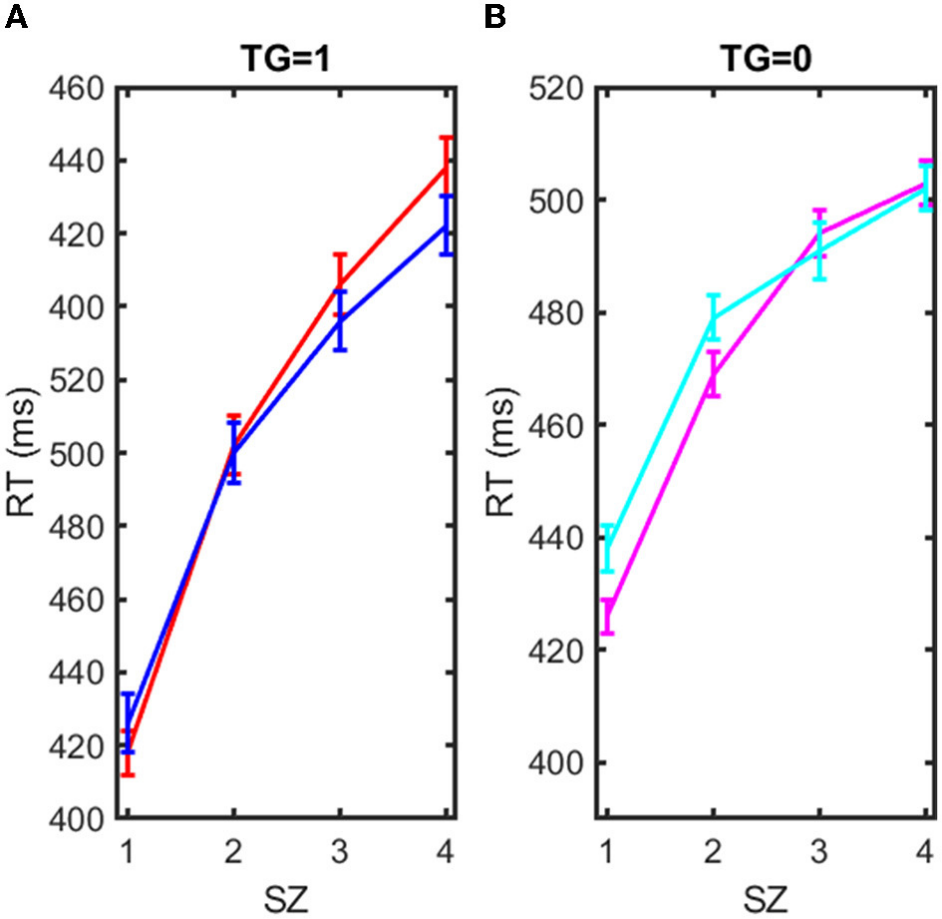
Representation of reaction time (RT) in milliseconds vs. the set of items to recall (SZ = 1, 2, 3, 4). **(A)** Blue solid line: RT mean vs. SZ for exposed EX = 1 and target case (TG = 1). Red solid line: RT mean vs. SZ for sham-exposed EX = 0 and target case (TG = 1). **(B)** Magenta solid line: RT mean vs. SZ for exposed EX = 1 and non-target case (TG = 0). Cyan solid line: RT mean vs. SZ for sham-exposed EX = 0 and non-target case (TG = 0).

The two groups presented excellent homogeneity, with hardly any differences between them. They were also homogeneous in the realization of errors and omissions. In the following, we selected the exposed individuals (EX = 1), and we calculated the average RT for TG = 1, and TG = 0 at each SZ = 1, 2, 3, 4. We did the same for the sham-exposed subjects (EX = 0). These means of RTs are shown in Table 4, where it is indicated in each case. The results of Table 4 are better explained in Figure 4, which shows the RTs vs. SZ with standard deviation for four cases, TG = 1 and EX = 0, TG = 1 and EX = 1, TG = 0 and EX = 0, and TG = 0, EX = 1. The plots show how RT is gradually decreasing with increasing SZ, which indicates a response acceleration (decreasing RTs) due to learning. Response acceleration is a common phenomenon in repeated response tests (Sternberg, 1975), and it has nothing to do with the EX or TG condition, as it is observed in both cases (EX = 0, 1 and TG = 0, 1). However, the scope of the results is best understood using the mathematical model of equation (2). The multi-regression model of equation (2) was applied to derive the parameters β_0_, β_1_, β_2_, β_3_ of equation (2) which provided the best fit to the RT data. These parameters have a statistical significance (*p* < 0.01) and are presented in Table 5.

**Table 4:** Mean values of the RTs in each case with their standard deviation, *p* < 0.01.

		**Exposed**	**Sham-exposed**
**TG**	**SZ**	**Mean (ms)**	**Mean (ms)**
1	1	399 (3)	403 (4)
1	2	441 (4)	440 (4)
1	3	463 (4)	458 (4)
1	4	479 (4)	471 (4)
0	1	426 (3)	438 (4)
0	2	469 (4)	479 (4)
0	3	494 (4)	491 (5)
0	4	503 (4)	502 (4)

**Table 5:** Multiple regression model (Equation 2): coefficients, standard error, *t*-value, and *p*-value of statistical association.

**Variable**	**Value (ms)**	**Std deviation (ms)**	***p*-value**
β_0_ (Intercept)	428.0	3.7	*p < * 0.001
β_1_ (SZ)	19.4	1.3	*p < * 0.001
β_2_ (EX)	−15.0	5.0	*p < * 0.002
β_3_ (SZ.EX)	6.3	1.8	*p < * 0.001
β_4_ (TG)	−29.0	2.0	*p < * 0.001

## 5. Discussion

### 5.1. Mathematical results

Both groups, exposed (EX = 1) and sham-exposed (EX = 0) are homogeneous; there are hardly any differences between the two groups in their habits, health status, and age, as shown in Tables 1, 2. The means of the errors and omissions made by both groups are almost identical, as shown in Table 3, although the difference is not significant (*p* > 0.05). Errors and omissions, with our data, have no statistically significant relationship with the exposure condition. However, our objective is not to study the errors made, we look for response delays, which are related to the functioning of WM. These delays are explained by the multi-regression of RTs, TG, EX, and SZ to provide the parameters β_0_, β_1_, β_2_, β_3_ of Equation (2).

Table 5 shows the results of the multi-regression adjustment, performed with Matlab^TM^, according to the mathematical model of Equation (2) using the data of the experiment, RTs, TG, EX, and SZ. The mean values of the RTs in each case are also shown in Table 4, and it is observed that for SZ = 1, one item to remember, RT is lower in the exposed group. However, this difference gradually decreases with the number of items (SZ), and the RT error, which is in the order of 3–4 ms, overlaps the results, and it is difficult to appreciate the difference. Therefore, the results are better appreciated with the linear multi-regression adjustment performed based on the mathematical model of Equation (2) shown in Table 5:

β_0_ = (428.1 ± 3.7) ms: The factor β_0_ is the zero intercept. β_0_ is the latency or minimum reaction time from stimulus to motor action in a cognitive task, is approximately 400 ms in young adults (Sternberg, 1975; Begleiter et al., 1993; Gladwin and Vink, 2021). This time interval is the same for everyone in both groups, regardless of condition, in the EX = 0, EX = 1, and the TG = 0, TG = 1. This time interval is the sum of two time intervals:

- The elapsed time from the moment the symbol appears on the screen until the search stimulus is triggered in the short-term memory part of the brain.
- The time that elapses from the instant the brain decides against pressing the mouse until the motor response of pressing the mouse is executed.

β_1_ = (19.4 ± 1.3) ms: The brain does serial scanning to find the presented symbol in the WM; in doing so, it spends β_1_ = (19.4 ± 1.3) ms per item, i.e., the scanning is linearly dependent on the number of symbols (SZ) to be remembered. This time depends on the health status and age of the subjects (Cascella and Al Khalili, 2022). Because the groups are highly homogeneous, it has a high significance *p* < 0.001.

β_4_ = (−29 ± 2) ms: If the screen symbol does not belong to the memorized set (TG = 0), it spends more time because it scans in the WM the time β_1_. SZ, until it is noticed that the symbol is not there, this WM operation takes extra time β_4_, in absolute value. Therefore, when the item is TG = 1, the RT is reduced by β_4_ = (−29 ±2) ms with respect to the case TG = 0. This time is the same for everyone in both groups, regardless of the condition, EX = 0, EX = 1, and S Z = 1, 2, 3, and 4.

β_2_ = (−15.4 ±4.9) ms: This is one of the most outstanding factors of the experiment. β_2_ has a negative value (*p* < 0.002) and is introduced in the mathematical model to account for the exposure (EX = 1). It has not been described or explained elsewhere, it is the first time that is accounted for. It means that the overall reaction time decreases with exposure by ~β_2_ = (−15.4 ± 4.9) ms. In the case of exposure to MF, this time is subtracted from β_0_, which is the time spent in perception and motor response described above. It means that exposure (EX = 1) reduces this time by 15 ms. The exposure reduces the RT response time by generating a kind of alert state, which is not captured by the senses, and which would be generated at the level of neurotransmitters in the prefrontal brain area of the brain. Magnetic stimulation would act similarly to transcranial stimulation (Fregni et al., 2005; Ikeda et al., 2019; Figeys et al., 2023), speeding up the movement of neurotransmitters in the alert state of the individual, and decreasing RT by 3.6%.

β_3_ = (6.3 ± 1.8) ms: This is the most outstanding factor of the experiment. The β_3_ factor is positive. The β_3_ factor relates RT to SZ through exposure (EX = 1). When there is MF exposure, EX = 1, and the response time increases β_3_ for each SZ. That indicates that with MF exposure, RT increases by 6.3 ms for searching and recalling one memory item, 12.6 ms for searching and recalling two memory items, and ... *n* x β_3_ for recalling *n* memory items. This would indicate a substantial degradation of the search process in WM. This degradation of WM, linearly related to the number of items to recall would materialize with an increase in RT.

The factor β_3_ has to be analised in the context of the value of β_1_. The ratio β_3_ /β_1_ = 0.32. In the model of equation (2) they appear together multiplying SZ relating to RT the effect of exposure with the search in WM: (β_1_+β_3_·EX)·SZ. From the results of our experiment the effect of MF exposure would result in a delay in the search time inside WM. The search time would increase by more than 30%: ΔRT = (β_1_+β_3_)·SZ.~1.32·β_1_·SZ.

Factors β_2_ and β_3_ should not be confused; β_3_ is related to the peripheral motor response, and β_2_ is related to the cognitive activity of the brain. From our results, perception and motor response are faster with magnetic stimulation because RT decreases with β_2_, but cognitive activity slows down because RT increases with β_3_. This is the interest of this study, which allows us to analyze both changes. On the one hand, the mathematical model to analyze the effect of the stimulus, which can be extended to any type of stimulus, and the way the model separates the effect between peripheral activity and cognitive activity of the brain, i.e., the serial scanning in the WM.

### 5.2. Biophysical mechanism

The MF induces noise in neural circuits and affects neurotransmitters, preventing the normal functioning of memory search. This is a substantial impairment of WM, which should be explained following a model. Memory impairment may be associated with a dysfunction of the synaptic neurotransmitters or with an alteration of the neuronal network that maintains memory.

An indirect reason for search delays may be due to the destruction of part of the neural network that supports WM. Destruction of the neuronal circuit is possible if the magnetic field is able to modify the functioning of the microglia indirectly by allowing the action of substances present outside of the membranes due to stress (Pall, 2013). Microglia are capable of phagocytosing dendrites that are activated during a cognitive process, and they typically phagocytose dead cells and debris in the brain (Hanisch and Kettenmann, 2007; Kettenmann et al., 2011; Ransohoff and Brown, 2012). They play key roles in the building of neurological circuits (Schafer et al., 2012), synaptic pruning (Paolicelli et al., 2011), neurogenesis (Sierra et al., 2010; Gemma and Bachstetter, 2013), and the clearance of hazardous factors (Ransohoff and Perry, 2009; Sierra et al., 2014; Tay et al., 2016). In contrast, the ability of the magnetic field to open the blood–brain barrier has been demonstrated previously, and the opening could permit some external substances to be actuated (Persson et al., 1997; Schirmacher et al., 2000; Leszczynski et al., 2002; Nittby et al., 2008; Tang et al., 2015). This is a hypothesis that should be validated in a future *in vivo* experiment.

The interaction of the magnetic field with spin generates unpaired states in chemical reactions (Lubitz et al., 2002). This mechanism is postulated to justify the sense of magnetic orientation that many birds have to guide them in migrations, (Ritz et al., 2004; Rodger and Hore, 2009). The magnetic effect on unpaired electron pairs, spin, is not excluded (Wiltschko et al., 2021), although it is difficult to find the target in this case.

The cyclotron resonant interaction, postulated by Liboff et al. (1987), Liboff (2019), is the oldest of the existing explanations of any biophysical mechanism. Cyclotron resonance is produced by the combination of a static magnetic field, the earth's magnetic field, with a time-varying one, which would generate a resonance that would modify the movement of ions Na^+^, K^+^, and Ca^+2^ through the cell membrane; however, for the present case, it is not convincing because of the low power of the MF (Zhadin et al., 1999).

The simplest possible explanation for these delays would be a direct effect of the magnetic field on the synaptic neurotransmitters, or areas of the mitochondria with a high density of charge carriers, the prefrontal cortex contains more mitochondria than other cortical regions (Hara et al., 2014), or memory engrams (Rao-Ruiz et al., 2021) whose activity would be modulated based on the force induced by the magnetic field (Esparza-Moltó et al., 2021). Ionized substances interact with magnetic fields due to the Lorentz force (**F** = q**v** x **B**), which acts on a moving charged particle (Stratton, 1941; Binhi and Prato, 2018). This interaction generates an induced electric field (**E** = -**v** x **B**) (Kraus, 1999), where **v** is the velocity of the particles. This interaction supports the Hall effect that occurs in conductive and semiconductor materials (Capasso, 1990). This phenomenon also occurs in the flow of electrolytes, e.g., within the circulatory system, and in the propagation of nerve impulses. This effect is measurable *in vivo* EEGs, (Bell et al., 1992; Freude et al., 1998; Glaser et al., 1998). The magnetic field would cause the neurotransmitters to rotate in the direction of the eddy currents, hindering their path and reducing their flow. The effect of increasing conductivity without favoring any specific circuit in the brain tissue would modulate the function of neurotransmitters and would generate a small amount of confusion, delaying the cognitive process. This phenomenon seems to be present in brain stimulation with electrical currents (Fregni et al., 2005; Ikeda et al., 2019; Murphy et al., 2020; Figeys et al., 2023). The magnetic field would act through eddy currents, the process by which induction kitchens work. Eddy currents rotate around the magnetic field lines, heating the biological tissue by the Joule effect. This is due to the conductivity of the cells, which have an electrical conductivity of σ~5 Siemens/meter. This slight heating makes them more sensitive to the substances present in the plasma, as has been demonstrated with other similar therapies in which physical stimulation is combined with drugs (Vanakoski and Seppälä, 1998; Mirvakili and Langer, 2021).

The power per unit mass that heats these cells is approximately:

P = π^2^.B^2^.d^2^.f^2^/(6. ρ.D) (W/Kg) (3)

B = 0.1x10^−6^ T;

d ~ 0.01m; we assume a thickness of the tissue under the scalp of ~1 cm.

f ~ 10 x 10^3^ Hz; frequency of B

ρ = 0.2 Ω.m; resistivity of tissue, ρ = 1/σ, where σ ~ 0.5 S/m approaches the tissue conductivity.

D ~ 1,000 kg/m^3^; tissue density

The power per mass kilogram is P ~ 8 x 10^−13^W/Kg.

This provides a very small amount of power, but at the cell level, it may be enough to enhance substance uptake. Alternatively, if there is insufficient power dissipation through blood pumping, it could result in tissue death by heating to ~45°, (Tenforde and Liburdy, 1988). In this sense, the effect would be similar to that obtained with an adjuvant (Mirvakili and Langer, 2021).

## 6. Conclusion

In this article, we have presented a mathematical model and an experimental procedure to study cognitive effects on WM. The model distinguishes between the effects on peripheral activity and cognitive activity in the brain. This model is simple and applicable to the non-invasive study of cognitive impairment under the action of stimuli of different origins. This procedure has been applied to the study of the effects of audio frequency magnetic stimulus on the area of the brain that supports WM. The procedure has been applied to a homogeneous set of 65 young, healthy subjects, i.e., undergraduate students. Tests performed with exposed and non-exposed groups have been analyzed and show statistically significant differences in response times that can be separated according to the mathematical model. The differences show a small reduction in the time spent in response selection, preparation, and execution of 15 ms over 428 ms (1.5%), the effect of which could resemble the activation of an alert situation. However, exposure results in 32% longer delays in memory search than in the case of non-exposure. These results indicate that exposure to magnetic fields in the audio frequency band could produce significant cognitive impairment in the young population. Finally, we believe that eddy currents play a plausible role in the biophysical mechanism of action that could be related to some physical stimulus or tissue micro heating.

## Data availability statement

The original contributions presented in the study are included in the article/supplementary material, further inquiries can be directed to the corresponding author.

## Ethics statement

The studies involving human participants were reviewed and approved by Comite de ética Universitat de València. The patients/participants provided their written informed consent to participate in this study.

## Author contributions

EAN: conceptualization, methodology, and validation. EN-M: software, data analysis, and graphics. EAN and EN-M: writing. All authors have read and agreed to the published version of the manuscript.
